# 

**Published:** 1905-07

**Authors:** 


					﻿Sixtieth Year.
JULY. 1905.
BUFFALO «•*
MEDICAL JOURNAL
VOL. LX.
Whole Number,
DCCIV.
vol. 3Kiv~-
Established 1845 by AUSTIN FLINT, M. D.
EDITOR :
WILLIAM WARREN POTTER, M. D.
ASSISTANT EDITORS:
■WILLIAM CT’RTTATTSS, M. D.
NELSON W. WILSON, M. D.
ASSOCIATE EDITORS:
JAMES WRIGHT PUTNAM, M. D.
JOHN PARMENTER, M. D.
HARVEY R. GAYLORD, M. D.
ERNEST WENDE, M. D.
JOHN A. MILLER, Ph. D.
MAUD J. FRYE, M. D.
				

